# Add-on sirolimus in refractory iMCD-TAFRO: a TAFRO-enriched 8-patient case series with paired single-cell profiling

**DOI:** 10.3389/fimmu.2026.1963440

**Published:** 2026-10-16

**Authors:** Remi Sumiyoshi, Tomohiro Koga, Toru Michitsuji, Masataka Umeda, Ayuko Takatani, Yoshito Nishimura, David C Fajgenbaum, Frits van Rhee, Atsushi Kawakami

**Affiliations:** 1 Department of Immunology and Rheumatology, Division of Advanced Preventive Medical Sciences, Nagasaki University Graduate School of Biomedical Sciences, Nagasaki, Japan; 2 Clinical Research Center, Nagasaki University Hospital, Nagasaki, Japan; 3 Division of Hematology, Mayo Clinic, Rochester, MN, United States; 4 Division of Translational Medicine and Human Genetics, University of Pennsylvania, Philadelphia, PA, United States; 5 Myeloma Center, Winthrop P. Rockefeller Cancer Institute, University of Arkansas for Medical Sciences, Little Rock, AR, United States; 6 DEJIMA Infectious Disease Research Alliance (DIDA), Nagasaki University, Nagasaki, Japan; 7 Biomedical Research and Career Support Office, Nagasaki University, Nagasaki, Japan

**Keywords:** castleman disease, idiopathic multicentric castleman disease, mTOR, refractory disease, single-cell RNA sequencing, sirolimus, TAFRO syndrome

## Abstract

**Introduction:**

Anti–IL-6 therapy with tocilizumab (TCZ) is ineffective in a proportion of patients with idiopathic multicentric Castleman disease (iMCD), particularly the TAFRO subtype, and rescue options remain limited. Evidence for mTOR inhibition comes mainly from cohorts in which iMCD-TAFRO was underrepresented.

**Methods:**

Eight Japanese patients with refractory iMCD (iMCD-TAFRO, n=6; iMCD-NOS, n=1) or TAFRO syndrome without iMCD (n=1) received add-on sirolimus (1–2 mg/day). Five had active refractory iMCD-TAFRO at initiation and formed the efficacy set; Case 5 (sirolimus for glucocorticoid sparing), Case 1 (iMCD-NOS) and Case 3 (TAFRO syndrome without iMCD) are reported descriptively. Response was defined as improvement in at least one refractory feature (CRP <0.3 mg/dL, platelets >15×10^4^/µL, improvement of anasarca, or a ≥50% rise in eGFR) at 3 and 12 months.

**Results:**

In the efficacy set, 4/5 responded at 3 months (80%; 95% CI 28–99) and 3/4 evaluable at 12 months (75%; 95% CI 19–99); a prespecified TAFRO-phenotype analysis (n=6, including Case 3) gave 5/6 (83%; 95% CI 36–100) and 4/5 evaluable (80%; 95% CI 28–99). Under a prespecified stringent definition additionally requiring CRP normalization and no escalation of concomitant immunosuppression, proportions fell to 3/5 (60%) and 2/4 evaluable (50%), each reclassification resting on a single criterion. All six responders eventually discontinued sirolimus without relapse during available follow-up. Glucocorticoids were withdrawn in all four responders receiving prednisolone, TCZ intervals were extended to ≥5 weeks in three patients, and both patients with dialysis-dependent active disease stopped hemodialysis. Exploratory paired single-cell RNA sequencing in one TAFRO-phenotype patient showed reductions in inflammatory monocytes and B cells with lower cytotoxic and proliferative programs.

**Discussion:**

Given the very small sample, wide confidence intervals and the sensitivity of the estimates to the response definition, add-on sirolimus should be regarded as a hypothesis-generating adjunctive rescue option on top of standard treatment, not a substitute, in refractory iMCD-TAFRO.

## 1 Introduction

The TAFRO subtype of idiopathic multicentric Castleman disease (iMCD-TAFRO) and TAFRO syndrome are severe inflammatory disorders that may cause life-threatening anasarca, thrombocytopenia, and renal failure requiring dialysis. Idiopathic multicentric Castleman disease (iMCD) is classified, on the basis of clinical and pathological features, as idiopathic plasmacytic lymphadenopathy (iMCD-IPL), iMCD-TAFRO, or iMCD-not otherwise specified (iMCD-NOS) (1, 2). TAFRO syndrome without iMCD is a related clinical entity that can be diagnosed even without definitive Castleman disease histopathology (3).

Interleukin-6 (IL-6)-directed therapy with tocilizumab (TCZ) or siltuximab is recommended as first-line treatment for iMCD in the Castleman Disease Collaborative Network (CDCN) consensus guidelines. (1, 4) Nevertheless, approximately one-third of patients, especially those with iMCD-TAFRO, respond inadequately to anti–IL-6 therapy, implicating IL-6-independent inflammatory pathways. (1, 5) Recent studies further suggest that iMCD-TAFRO is characterized by subtype-specific endothelial IL-6 production and activation of additional inflammatory pathways, including TNF signaling, highlighting a complex inflammatory network that extends beyond IL-6 alone. (6, 7) Rescue therapy often requires prolonged glucocorticoids or empiric immunosuppression, both of which have substantial limitations.

Activation of the phosphatidylinositol-3-kinase (PI3K)/Akt/mammalian target of rapamycin (mTOR) pathway has been implicated in refractory iMCD. (5, 8) Increased phospho-S6, phospho-4E-BP1, and phospho-p70S6K expression in iMCD lymphoid tissue, together with proteomic enrichment of mTORC1-related pathways, supports mTOR signaling as a therapeutic target. (9) Sirolimus, an mTOR inhibitor, is therefore of clinical interest. Preliminary evidence from a case report and a retrospective study of 26 patients with refractory/relapsed iMCD suggests potential efficacy, with 18 patients achieving symptomatic and biochemical responses in the retrospective cohort. (10, 11) A Japanese investigator-initiated placebo-controlled trial of sirolimus in refractory iMCD ended early because of recruitment and logistical difficulties, leaving only limited descriptive data. (12, 13) Other off-label approaches, including ruxolitinib, thalidomide, cyclosporine A, and anakinra, have also been used, but evidence remains sparse. (14, 15) Alternative targeted approaches are also emerging: a 52-week phase Ib study of the JAK1-preferential inhibitor filgotinib and a retrospective study of the BTK inhibitor orelabrutinib provided preliminary evidence in iMCD, although no optimal rescue strategy has been established. (16, 17).

Patients with TAFRO manifestations have been underrepresented in prior sirolimus reports. (10) This is clinically important because the TAFRO-subtype is often marked by severe thrombocytopenia, anasarca, renal dysfunction, and inadequate anti–IL-6 responses. Real-world experience with sirolimus in TAFRO-enriched populations, particularly among Japanese patients, remains limited. Longitudinal immune profiling during sirolimus treatment in iMCD has been reported at the level of serum proteomics, (18) but cell-type-resolved profiling of the peripheral immune compartment before and during sirolimus treatment has not, to our knowledge, been described.

Here, we report eight consecutive patients with refractory iMCD-TAFRO, iMCD-NOS, and TAFRO syndrome without iMCD who received add-on sirolimus. We also performed exploratory paired single-cell RNA sequencing (scRNA-seq) of peripheral blood mononuclear cells from one representative TAFRO-phenotype patient (TAFRO syndrome without iMCD) at diagnosis before any treatment and 3 months after add-on sirolimus initiation, while the patient was also receiving tocilizumab and prednisolone. This analysis was designed to illustrate treatment-associated immune changes rather than to support statistical inference. Our aims were to assess the clinical feasibility of add-on sirolimus in refractory iMCD-TAFRO and to generate hypotheses about associated immune changes.

## 2 Materials and methods

### 2.1 Patients and treatment

Between April 2018 and December 2024, eight consecutive patients with idiopathic multicentric Castleman disease (iMCD) or TAFRO syndrome at Nagasaki University Hospital received add-on sirolimus (1–2 mg/day) for disease activity that remained inadequately controlled despite standard treatment. Use of sirolimus was approved by the institutional off-label drug-use committee. The study was approved by the institutional review board of Nagasaki University (approval number 22031401), and all participants provided written informed consent. One additional patient treated under the same protocol was excluded after subsequent histopathological reclassification as B-cell lymphoma.

Castleman disease (CD) was diagnosed according to Japanese diagnostic criteria, (19) and TAFRO syndrome according to the updated Masaki 2019 criteria. (20) All patients classified as iMCD-TAFRO or as TAFRO syndrome without iMCD fulfilled these diagnostic criteria at the time of diagnosis; the clinical data presented in this report describe the patients at sirolimus initiation, at which point individual features had in some patients already been modified by prior therapy. Histopathological findings were reviewed by board-certified pathologists of the Japanese Society of Pathology and classified as plasma cell, hypervascular/hyaline vascular, or mixed type.

Clinical subtypes of iMCD (iMCD-TAFRO, iMCD-IPL, and iMCD-NOS) were determined by consensus between two board-certified rheumatologists (T.K. and R.S.) affiliated with national research programs for Castleman disease and TAFRO syndrome (Study Group from the Ministry of Health, Labour and Welfare, Japan), according to previously described definitions. (3, 19, 20) Cases presenting with thrombocytopenia, anasarca, fever, reticulin fibrosis, and organomegaly were classified as iMCD-TAFRO; cases with serum IgG levels >3500 mg/dL and lymph node histology showing germinal-center hyperplasia with sheet-like plasma-cell proliferation were classified as iMCD-IPL; all remaining cases were classified as iMCD-NOS.

Inadequate response to prior therapy was defined as persistent CRP elevation (≥1 mg/dL), thrombocytopenia (platelet count <10×10^4^/µL), persistent anasarca, or progressive renal dysfunction despite TCZ-based treatment, with or without glucocorticoids, calcineurin inhibitors, or rituximab. All eight enrolled patients were included in the safety evaluation. Efficacy was prespecified to be evaluated in the patients with active refractory iMCD-TAFRO at sirolimus initiation. Case 5 received sirolimus mainly for glucocorticoid sparing in clinically inactive disease (baseline CRP, 0.14 mg/dL; normal platelet count; normal renal function), and was excluded from the efficacy analysis but included in the safety evaluation. Case 1 (iMCD-NOS) and Case 3 (TAFRO syndrome without iMCD) were reported descriptively and excluded from the iMCD-TAFRO efficacy analysis. A prespecified secondary analysis combined the iMCD-TAFRO patients with Case 3 to form a broader TAFRO-phenotype group. Throughout this report, “TAFRO phenotype” denotes the shared clinical presentation of iMCD-TAFRO and TAFRO syndrome without iMCD; it is not used as a diagnostic category, and it is applied only to this prespecified exploratory secondary analysis and to the single patient in whom single-cell profiling was performed. Disease activity was assessed at baseline and during follow-up using serum CRP, platelet count, creatinine, hemoglobin, albumin, the CHAP composite score, and the TAFRO severity score. The CHAP score is the Japanese Castleman disease activity composite, which grades CRP, hemoglobin, albumin and performance status from 0 to 3 each for a total of 0–12, with higher scores indicating more active disease. (19) The TAFRO severity score is the four-item disease severity classification of the updated Masaki 2019 criteria, (20) which grades anasarca, thrombocytopenia, fever/inflammation and renal dysfunction from 0 to 3 each for a total of 0–12.

A clinically meaningful response was defined as improvement in at least one refractory feature that was present at sirolimus initiation: CRP normalization (<0.3 mg/dL), platelet recovery (>15×10^4^/µL), improvement or resolution of anasarca on clinical examination or imaging, or renal improvement. Following peer review, the renal criterion was redefined directionally as a relative increase of at least 50% in the estimated glomerular filtration rate (eGFR) from the pre-sirolimus baseline, or withdrawal from renal replacement therapy, in place of the original non-directional threshold of a serum creatinine below 1.5-fold baseline. eGFR was chosen over creatinine because it is adjusted for age and sex and is less sensitive to the changes in muscle mass and fluid balance that accompany recovery from active TAFRO-phenotype disease. The renal criterion is evaluated only in the patients who had renal dysfunction at sirolimus initiation, defined as an eGFR below 60 mL/min/1.73 m² or a requirement for renal replacement therapy. Reanalysis under the directional criterion did not reclassify any patient at either timepoint (**Table 1**).

**Table 1:** Per-patient, criterion-by-criterion response assessment at 3 and 12 months under the primary and stringent definitions.

Case	Subtype	Analysis cohort	Time- point	On sirolimus	CRP <0.3 mg/dL	Platelets >15×10^4^/µL	Anasarca (score/baseline)	Renal improvement (≥50% ↑ eGFR)	No escalation of immunosuppression	Response (primary)	Response (stringent)
1	iMCD-NOS	Descriptive	3 mo	Yes	Not met, 0.37	Met, 26.0	n/a	n/a	Yes	Non-responder	Non-responder
			12 mo	Yes	Met, 0.17	Met, 30.4	n/a	n/a	Yes	Responder	Responder
2	iMCD-TAFRO	Efficacy	3 mo	Yes	Not met, 2.44	Not met, 5.7	n/a	n/a	Yes	Non-responder	Non-responder
			12 mo	No	Not met, 2.42	Not met, 7.4	n/a	n/a	Yes	Non-responder	Non-responder
3	TAFRO w/o iMCD	Secondary	3 mo	Yes	Met, 0.25	Not met, 10.8	Met, 2/3	n/a	Yes	Responder	Responder
			12 mo	Yes	Met, 0.03	Met, 31.0	Met, 0/3	n/a	Yes	Responder	Responder
4	iMCD-TAFRO	Efficacy	3 mo	Yes	Met, 0.01	Met, 29.2	Met, 2/3	Met, 50/14 (+250%)	Yes	Responder	Responder
			12 mo	Yes	Met, 0.03	Met, 30.9	Met, 0/3	Met, 54/14 (+276%)	Yes	Responder	Responder
5	iMCD-TAFRO	Safety only	3 mo	No	n/a	n/a	n/a	n/a	Yes	Not applicable	Not applicable
			12 mo	No	n/a	n/a	n/a	n/a	Yes	Not applicable	Not applicable
6	iMCD-TAFRO	Efficacy	3 mo	Yes	Met, 0.26	Met, 22.7	Not met, 3/3	Met, 54/29 (+89%)	Yes	Responder	Responder
			12 mo	Yes	Not met, 0.56	Met, 26.2	Met, 0/3	Met, 45/29 (+57%)	Yes	Responder	Non-responder
7	iMCD-TAFRO	Efficacy	3 mo	Yes	Met, 0.01	Met, 25.4	Met, 2/3	Met, 56/18 (+206%)	Yes	Responder	Responder
			12 mo	No	Met, 0.01	Not met, 13.8	Met, 1/3	Met, 50/18 (+173%)	Yes	Responder	Responder
8	iMCD-TAFRO	Efficacy	3 mo	No	Met, 0.02	Met, 16.5	Not met, 3/3	Met, 77/19 (+305%)	No	Responder	Non-responder
			12 mo	No	NE	NE	NE	NE	NE	NE	NE

Primary response definition: improvement in ≥1 refractory feature present at sirolimus initiation (CRP <0.3 mg/dL, platelets >15×10^4^/µL, improvement or resolution of anasarca, or renal improvement). Stringent (sensitivity) definition: CRP normalization (<0.3 mg/dL) AND platelet recovery and/or improvement in renal function or anasarca AND no escalation of concomitant immunosuppressive therapy after sirolimus initiation.

Values are the observed measurement at the visit closest to the nominal timepoint. Anasarca is shown as the anasarca sub-score of the TAFRO severity score at the timepoint over the baseline value (“Met”, decrease from baseline). Renal improvement is assessed only in patients with renal dysfunction at initiation and is shown as the eGFR at the timepoint over the pre-sirolimus baseline with the percentage change (“Met”, increase of ≥50% or withdrawal from renal replacement therapy).

“On sirolimus” indicates whether the patient was still receiving sirolimus at that assessment (shaded, No).

n/a, criterion not applicable because the feature was absent or normal at baseline; NE, not evaluable; eGFR, estimated glomerular filtration rate. Case 5 was clinically inactive at initiation and contributes to the safety cohort only.

Shaded cells in the response columns indicate discordance between the primary and stringent classifications (Case 8 at 3 months, Case 6 at 12 months); the reasons are given in the text.

Response was assessed at 3 and 12 months after sirolimus initiation in the patients with active iMCD-TAFRO. Anasarca was operationalized as the anasarca sub-score of the Japanese TAFRO syndrome severity classification, with improvement defined as any decrease from the baseline sub-score and resolution as a sub-score of zero. A prespecified sensitivity analysis applied a more stringent definition requiring all of the following: (1) CRP normalization (<0.3 mg/dL); (2) platelet recovery and/or improvement in renal function or anasarca; and (3) no escalation of concomitant immunosuppressive therapy after sirolimus initiation. Eltrombopag was not counted as immunosuppressive therapy, and rituximab infusions given shortly after sirolimus initiation as continuations of courses begun beforehand were not counted as escalation. Every component of both definitions is reported for every patient at both timepoints in **Table 1**. Because several patients discontinued sirolimus before a nominal assessment, each assessment is additionally labeled according to whether the patient was still receiving sirolimus at that visit (**Tables 1**, **2**), so that disease status at a fixed time after initiation is not confused with response during continuing exposure. We also evaluated durability of response and treatment-sparing outcomes, including prednisolone discontinuation, extension of TCZ dosing intervals, and withdrawal from hemodialysis. Concomitant TCZ, prednisolone, rituximab, and cyclosporine A were continued at the treating physicians’ discretion and were not modified by protocol.

**Table 2:** Treatment course, disease activity and concomitant therapy at sirolimus initiation and at 3 and 12 months.

Case	Subtype	Time- point	Sirolimus status	CRP (mg/dL)	Platelets (×10^4^/µL)	Creatinine (mg/dL)	Albumin (g/dL)	CHAP score	TAFRO score	Prednisolone (mg/day)	Tocilizumab interval	Other concomitant therapy	Renal replacement therapy
1	iMCD-NOS	0 mo	On sirolimus (initiation)	1.17	27.0	0.95	4.4	1	—	None	q2w	—	No
		3 mo	On sirolimus	0.37	26.0	0.94	4.4	0	—	None	q2w	—	No
		12 mo	On sirolimus	0.17	30.4	0.92	4.8	0	—	None	q2w	—	No
2	iMCD-TAFRO	0 mo	On sirolimus (initiation)	2.52	8.2	10.73	4.1	1	5	None	q1w	—	Yes
		3 mo	On sirolimus	2.44	5.7	11.89	3.8	1	5	None	q1w	—	Yes
		12 mo	Off sirolimus (stopped 3.7 mo)	2.42	7.4	9.77	4.2	1	5	None	q1w	—	Yes
3	TAFRO w/o iMCD	0 mo	On sirolimus (initiation)	8.62	3.1	0.57	2.0	9	6	25	q1w	ELT	No
		3 mo	On sirolimus	0.25	10.8	0.68	3.3	0	2	13	q2w	ELT	No
		12 mo	On sirolimus	0.03	31.0	0.73	3.9	1	0	5	q3w	—	No
4	iMCD-TAFRO	0 mo	On sirolimus (initiation)	4.77	7.8	3.95	3.3	4	8	None (60 from day 6)	q1w	—	No
		3 mo	On sirolimus	0.01	29.2	1.25	4.3	2	3	15	q1w	—	No
		12 mo	On sirolimus	0.03	30.9	1.17	4.5	0	1	6	q3w	—	No
5	iMCD-TAFRO	0 mo	On sirolimus (initiation)	0.14	26.2	0.54	—	0	0	10	q2w	CyA	No
		3 mo	Not evaluable	NE	NE	NE	NE	NE	NE	NE	NE	NE	NE
		12 mo	Not evaluable	NE	NE	NE	NE	NE	NE	NE	NE	NE	NE
6	iMCD-TAFRO	0 mo	On sirolimus (initiation)	0.43	11.3	1.91	2.7	4	5	None	—	RTX, ELT	No
		3 mo	On sirolimus	0.26	22.7	1.07	3.9	0	4	None	—	ELT	No
		12 mo	On sirolimus	0.56	26.2	1.26	4.2	0	1	None	—	—	No
7	iMCD-TAFRO	0 mo	On sirolimus (initiation)	11.63	2.5	2.89	2.4	11	10	60	q1w	ELT	Yes
		3 mo	On sirolimus	0.01	25.4	1.04	3.7	2	3	9	q2w	ELT	No
		12 mo	Off sirolimus (stopped 5.5 mo)	0.01	13.8	1.15	4.1	0	2	4	q4w	—	No
8	iMCD-TAFRO	0 mo	On sirolimus (initiation)	2.73	2.9	2.01	2.5	8	8	20	—	RTX, ELT	Yes
		3 mo	Off sirolimus (stopped 1.6 mo)	0.02	16.5	0.56	2.7	6	3	0	q2w	ELT	No
		12 mo	Not evaluable	NE	NE	NE	NE	NE	NE	NE	NE	NE	NE

Assessments made after sirolimus discontinuation are shaded.

Values are the observed measurement at the visit closest to each nominal timepoint. Shaded “Off sirolimus” cells mark assessments made after sirolimus had been discontinued.

Prednisolone values are maintenance doses; methylprednisolone pulses are not shown. In Case 4, prednisolone was started 6 days after sirolimus initiation. Case 8 was not receiving tocilizumab at initiation (replaced by rituximab) and tocilizumab was reintroduced at 2.1 months. Rituximab is shown where an infusion was given within 1.5 months of the timepoint.

Case 5 was followed at another institution from 0.7 months on an unchanged prednisolone dose of 10 mg/day; Case 8 transferred to another institution at 3.0 months.

CyA, cyclosporine A; ELT, eltrombopag; RTX, rituximab; NE, not evaluable. Renal replacement therapy in Case 2 was chronic maintenance hemodialysis unrelated to Castleman disease.

Because whole-blood sirolimus therapeutic drug monitoring was not routinely available at our institution, efficacy was evaluated using clinical outcomes at fixed low doses (1–2 mg/day).

Prior publication of individual cases. To allow readers to judge how much independent evidence this series contributes, the prior-publication status of every patient is stated explicitly. Case 1 has been reported previously as a single-patient case report of tocilizumab-refractory iMCD successfully treated with sirolimus; (11) the present analysis uses updated follow-up extending well beyond the period described in that report. The lymph-node histopathology of Cases 2, 4, 6 and 7 was included, as validation cases, in the supplementary material of an international consensus study of histopathological subtyping of iMCD (21) that study listed the agents they had received but reported no treatment course or outcome data. Cases 3, 5 and 8 have not been reported previously in any form, and the paired peripheral single-cell dataset from Case 3 is reported here for the first time. No patient in this series participated in our investigator-initiated, placebo-controlled exploratory trial of sirolimus for tocilizumab-resistant iMCD or its long-term extension. (12, 13) The eight patients described here also do not include the patient whose longitudinal serum proteomic profile we reported separately. (18) The clinical course on add-on sirolimus of seven of the eight patients, and the entire single-cell dataset, are therefore reported here for the first time.

### 2.2 Statistical analysis

This is a descriptive case series and no formal comparative hypothesis testing was performed. Continuous variables are summarized as medians with ranges, and categorical variables as counts with the explicit denominator. Response proportions are reported as fractions with exact (Clopper–Pearson) 95% confidence intervals; because the denominators are four to six patients, these intervals are very wide and are presented so that readers can judge the precision of each estimate rather than as evidence of a treatment effect. Patients without an assessment within the ±3-month window around a nominal timepoint are reported as not evaluable and are excluded from the denominator at that timepoint rather than imputed; the number excluded is stated wherever a proportion is given. No imputation was performed for missing laboratory values. Statistical analyses were performed in Python 3.11 (SciPy 1.14).

### 2.3 Single-cell RNA sequencing

Peripheral blood samples were collected from Case 3 (a 36-year-old woman with TAFRO syndrome) at two timepoints. The first sample was obtained at diagnosis, during active disease and before any treatment had been started (treatment-naïve: before the methylprednisolone pulse and before any tocilizumab or sirolimus). The second sample was obtained at the 3-month visit after sirolimus initiation, on the same day as the 3-month clinical assessment, while the patient was receiving sirolimus (1 mg/day) together with tocilizumab (every 2 weeks) and tapering prednisolone (13 mg/day, from a starting dose of 50 mg/day); at that visit CRP had normalized and the patient was in clinical response. The two samples are labeled “active” and “sirolimus” throughout for brevity, but the differences between them reflect the combined effects of the whole treatment regimen (glucocorticoids, tocilizumab and sirolimus) and of clinical recovery, and cannot be attributed to sirolimus alone. Peripheral blood mononuclear cells (PBMCs) were isolated by Ficoll density-gradient centrifugation and processed using the Chromium Single Cell 3′ v3.1 platform (10x Genomics; approximately 10,000 cells/sample). Libraries were sequenced on an Illumina NovaSeq 6000 platform (paired-end 28 + 90 bp) and aligned to the GRCh38 reference genome using Cell Ranger v6.1.2.

Downstream analyses were performed using Scanpy v1.11.1 (22). Cells with fewer than 200 detected genes, more than 5,000 detected genes, more than 25,000 unique molecular identifiers, or more than 15% mitochondrial reads were excluded. After quality control, 14,839 cells (active disease, 6,251; sirolimus treatment, 8,588) were retained for analysis.

Preprocessing consisted of library-size normalization, logarithmic transformation, selection of 2,000 highly variable genes, regression of total counts and mitochondrial-read percentage, scaling, principal component analysis (30 components), construction of a k-nearest-neighbor graph (k=15 using 20 principal components), Uniform Manifold Approximation and Projection (UMAP), and Leiden clustering (resolution=0.5). Cell populations were annotated using canonical marker genes.

Treatment-associated transcriptional changes were evaluated by per-cell-type pseudobulk effect-size analysis and gene-level fold-change comparisons. Cell-wise pathway scores for mTOR signaling, cytotoxicity, and inflammatory programs were calculated using sc.tl.score_genes, and Hallmark pathway enrichment was analyzed with GSEApy.

Lineage-restricted analyses were performed for monocyte and B/plasma-cell populations. Monocytes were subclustered using the top 1,500 highly variable genes, principal component analysis (20 components), k-nearest-neighbor graph construction (k=10 using 15 principal components), UMAP visualization, and Leiden clustering (resolution=0.5).

Because the transcriptomic analysis used paired samples from a single patient, findings were interpreted as exploratory within-patient biological changes and not as formal patient-level statistical inference.

## 3 Results

### 3.1 Patient characteristics and clinical response

Eight patients (5 men and 3 women; median age, 47.5 years [range, 33–81]) received add-on sirolimus and constituted the full cohort (**Table 3**, **Figure 1**). One patient had iMCD-NOS, six had iMCD-TAFRO, and one had TAFRO syndrome without iMCD. We evaluated efficacy in the five patients who had active refractory iMCD-TAFRO at sirolimus initiation (Cases 2, 4, 6, 7, and 8). Case 5 (iMCD-TAFRO) was excluded from the efficacy analysis because sirolimus was started for glucocorticoid sparing in clinically inactive disease. Case 1 (iMCD-NOS) and Case 3 (TAFRO syndrome without iMCD) were reported descriptively. A prespecified secondary analysis combined the five iMCD-TAFRO patients with Case 3 to form a broader TAFRO-phenotype group (n=6). Safety was evaluated across all eight patients. At sirolimus initiation, six patients were receiving TCZ, including four who were also receiving prednisolone; two were receiving rituximab, and one was also receiving cyclosporine A. Across the eight patients, the regimen in place at sirolimus initiation had been continued for a median of 2.8 months (range, 0.4–26.0 months) beforehand. In the six patients in whom that regimen was TCZ-based, the duration of TCZ exposure before sirolimus was 23.4, 26.0, 1.2, 4.4, 12.1 and 0.4 months in Cases 1, 2, 3, 4, 5 and 7, respectively; in Cases 6 and 8 tocilizumab had already been judged ineffective and had been replaced by rituximab before sirolimus was started (**Table 3**). Two patients (Cases 7 and 8) required hemodialysis for acute renal failure attributable to active disease. Case 2 was on chronic maintenance hemodialysis unrelated to Castleman disease and was excluded from analyses of dialysis-dependent disease activity.

**Table 3:** Baseline characteristics, prior and concomitant therapy, refractory features and disease-activity scores of the eight patients at sirolimus initiation.

Case	Age (y)	Sex	Clinical subtype	Lymph-node pathology	Analysis cohort	Therapy before sirolimus	Concomitant therapy at sirolimus initiation	Duration of that regimen before sirolimus (months)	Refractory features at sirolimus initiation	CRP (mg/dL)	Platelets (×10^4^/µL)	Creatinine (mg/dL)	Albumin (g/dL)	CHAP score	TAFRO score
1	44	M	iMCD-NOS	Plasma cell	Descriptive	Prednisolone, cyclosporine A	Tocilizumab	23.4	CRP elevation	1.17	27.0	0.95	4.4	1	—
2	49	M	iMCD-TAFRO	Plasma cell	Efficacy	None	Tocilizumab	26.0	CRP elevation; thrombocytopenia	2.52	8.2	10.73	4.1	1	5
3	36	F	TAFRO syndrome without iMCD	Not obtained	Secondary	Tacrolimus	Prednisolone, tocilizumab, eltrombopag	1.2	CRP elevation; anasarca; thrombocytopenia	8.62	3.1	0.57	2.0	9	6
4	46	M	iMCD-TAFRO	Plasma cell	Efficacy	Prednisolone	Tocilizumab (prednisolone from day 6)	4.4	CRP elevation; anasarca; thrombocytopenia; renal dysfunction	4.77	7.8	3.95	3.3	4	8
5	33	F	iMCD-TAFRO	Mixed	Safety only	None	Prednisolone, tocilizumab, cyclosporine A	12.1	None (inactive disease)	0.14	26.2	0.54	—	0	0
6	67	M	iMCD-TAFRO	Plasma cell	Efficacy	Prednisolone, tocilizumab	Rituximab, eltrombopag	0.4	Anasarca; renal dysfunction	0.43	11.3	1.91	2.7	4	5
7	64	M	iMCD-TAFRO	Hypervascular	Efficacy	None	Prednisolone, tocilizumab, eltrombopag	0.4	CRP elevation; anasarca; thrombocytopenia; renal dysfunction	11.63	2.5	2.89	2.4	11	10
8	81	F	iMCD-TAFRO	Plasma cell	Efficacy	Tocilizumab	Prednisolone, rituximab, eltrombopag	1.1	CRP elevation; anasarca; thrombocytopenia; renal dysfunction	2.73	2.9	2.01	2.5	8	8

Analysis cohorts: Efficacy, active refractory iMCD-TAFRO at sirolimus initiation (Cases 2, 4, 6, 7 and 8); Secondary, the efficacy set plus Case 3 (TAFRO phenotype); Descriptive, iMCD-NOS (Case 1); Safety only, clinically inactive disease at initiation (Case 5). All eight patients contribute to the safety analysis.

“Concomitant therapy at sirolimus initiation” is the regimen in place when sirolimus was added; “Duration of that regimen before sirolimus” is the interval from the start of that regimen to sirolimus initiation. In Case 4, prednisolone 60 mg/day was started 6 days after sirolimus initiation (shown in parentheses).

Laboratory values and scores are those at the pre-sirolimus assessment. The TAFRO severity score is not applicable to Case 1 (iMCD-NOS). In Case 6 the baseline CRP (0.43 mg/dL) was only marginally above the 0.3 mg/dL threshold and was not counted as a refractory feature. Only baseline values are available for Case 5, who was followed at another institution from 0.7 months.

M, male; F, female; iMCD, idiopathic multicentric Castleman disease; NOS, not otherwise specified; TAFRO, thrombocytopenia, anasarca, fever, reticulin fibrosis, organomegaly; CRP, C-reactive protein; CHAP, Castleman disease activity composite score.

**Figure 1:**
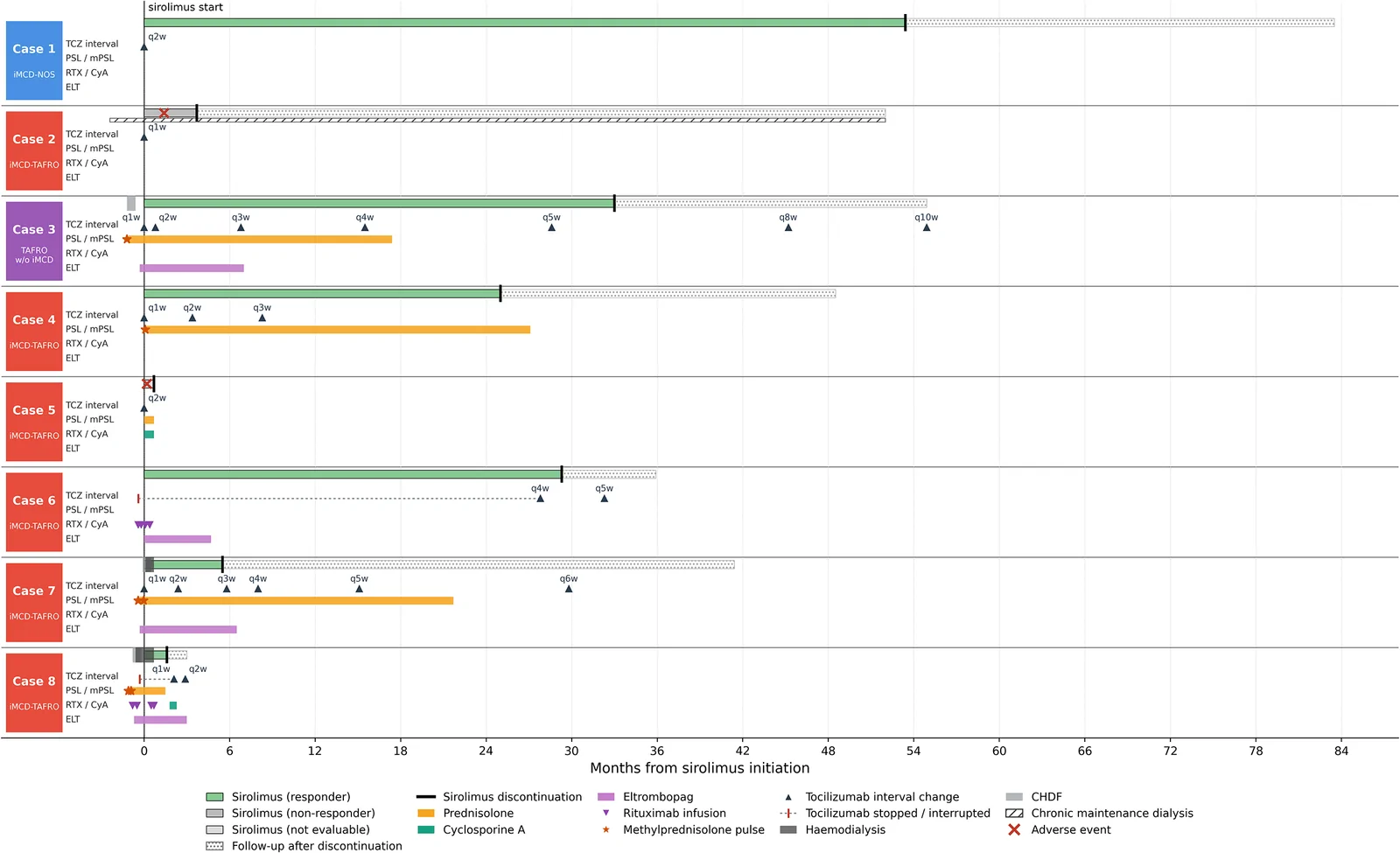
Individual treatment course and concomitant therapy on add-on sirolimus. Swimmer plot of the eight patients, one horizontal band per patient, aligned to the date of sirolimus initiation (month 0). Within each band the upper bar shows the duration of sirolimus exposure (green, responder; dark gray, non-responder; light gray, not evaluable), follow-up after sirolimus discontinuation (dotted) and the date of discontinuation (vertical black bar); hemodialysis (dark gray block), continuous hemodiafiltration (light gray block) and chronic maintenance hemodialysis (hatched strip; Case 2, unrelated to Castleman disease) are drawn on this bar, and adverse events during the observation period are marked (red ×). Concomitant therapy is shown in four labeled lanes beneath the bar: the tocilizumab dosing interval (triangles mark each change, labeled with the interval; a red tick and a dashed line mark the period during which tocilizumab was stopped, in Cases 6 and 8), prednisolone maintenance therapy (orange bar) with methylprednisolone pulses (stars), rituximab infusions (inverted triangles) and cyclosporine A (teal bar), and eltrombopag (violet bar). Events more than about 2 months before sirolimus initiation are not shown. The subtype of each patient is given in the label block: blue, iMCD-NOS; red, iMCD-TAFRO; purple, TAFRO syndrome without iMCD.

Among the five iMCD-TAFRO patients with active disease, 4 of 5 (80%; 95% CI 28–99; Cases 4, 6, 7, and 8) met the prespecified response criterion at 3 months. Case 2 was a non-responder and discontinued sirolimus at 3.7 months because of fever and lymphocytopenia. At 12 months, 3 of 4 evaluable patients (75%; 95% CI 19–99; Cases 4, 6, and 7) remained responders. Case 8 transferred to another institution at 3.0 months after elective sirolimus discontinuation at 1.6 months because of concern for possible invasive pulmonary aspergillosis; the patient was therefore not evaluable at 12 months but maintained response throughout available follow-up. Case 6 sustained clinical response despite a transient CRP increase at the 12-month visit (0.56 mg/dL), which subsequently normalized; under the stringent definition Case 6 therefore did not meet the CRP-normalization component at the 12-month timepoint (**Table 1**). Two of the four patients assessed at 12 months (Cases 2 and 7) had already discontinued sirolimus by that visit, as had Case 8 by the 3-month visit, so these assessments describe disease status at a fixed interval after initiation rather than response during continuing exposure; the treatment state at every assessment is given in **Table 2**. In the broader TAFRO-phenotype group (n=6, including Case 3), response rates were 5 of 6 (83%; 95% CI 36–100) at 3 months and 4 of 5 evaluable patients (80%; 95% CI 28–99) at 12 months. Given the small denominators, these confidence intervals span most of the plausible range and the point estimates should be interpreted with caution. Among the two descriptively reported cases, Case 1 (iMCD-NOS) showed a substantial CRP decrease (1.17 to 0.37 mg/dL at 3 months; 0.35 and 0.56 mg/dL at 6 and 9 months) and CRP normalization by 12 months (0.17 mg/dL), with no relapse during follow-up. Case 3 (TAFRO syndrome without iMCD) achieved CRP normalization within 3 months (8.62 to 0.25 mg/dL) and sustained response thereafter.

Longitudinal clinical findings are summarized in **Figure 2**. CRP decreased substantially in all responders, including both primary efficacy and descriptive cases, with normalization (<0.3 mg/dL) within 1–3 months in five patients and by month 12 in the remaining patient (Case 1) (**Figure 2A**). Platelet counts improved in patients with baseline thrombocytopenia (**Figure 2B**). Renal function improved in all four patients who had renal dysfunction attributable to active disease at sirolimus initiation (**Figure 2C**): eGFR rose from 14 to 50 mL/min/1.73 m² in Case 4, from 29 to 54 in Case 6, from 18 to 56 in Case 7 and from 19 to 77 in Case 8 by 3 months, corresponding to relative increases of 89% to 305%. Both patients with dialysis-dependent active disease (Cases 7 and 8) discontinued hemodialysis within 3 weeks after sirolimus initiation (Case 7 on day 9 and Case 8 on day 21, counting from the first dose of sirolimus to the last dialysis session). Case 2 remained on chronic maintenance hemodialysis for end-stage renal disease independent of Castleman disease throughout. Neither of the two patients whose renal function was normal at initiation developed renal impairment: eGFR never fell below 60 mL/min/1.73 m² in either patient, with a minimum of 63.9 in Case 1 over 83 months of follow-up and 67.2 in Case 3 over 55 months. Serum creatinine in Case 3 rose from 0.57 to 0.73 mg/dL over the first 12 months, but the baseline value was measured during severe hypoalbuminemia (albumin 2.0 g/dL), generalized fluid retention and muscle wasting and was therefore artefactually low; the rise accompanied nutritional and functional recovery (albumin 2.0 to 3.9 g/dL, hemoglobin 9.4 to 12.6 g/dL, anasarca sub-score 3 to 0, and body weight 61.0 kg while edematous, falling to 59.2 kg as edema resolved and then rising to 62.4 kg) rather than renal injury. This illustrates why the renal criterion is based on eGFR and is restricted to patients with renal dysfunction at baseline.

**Figure 2:**
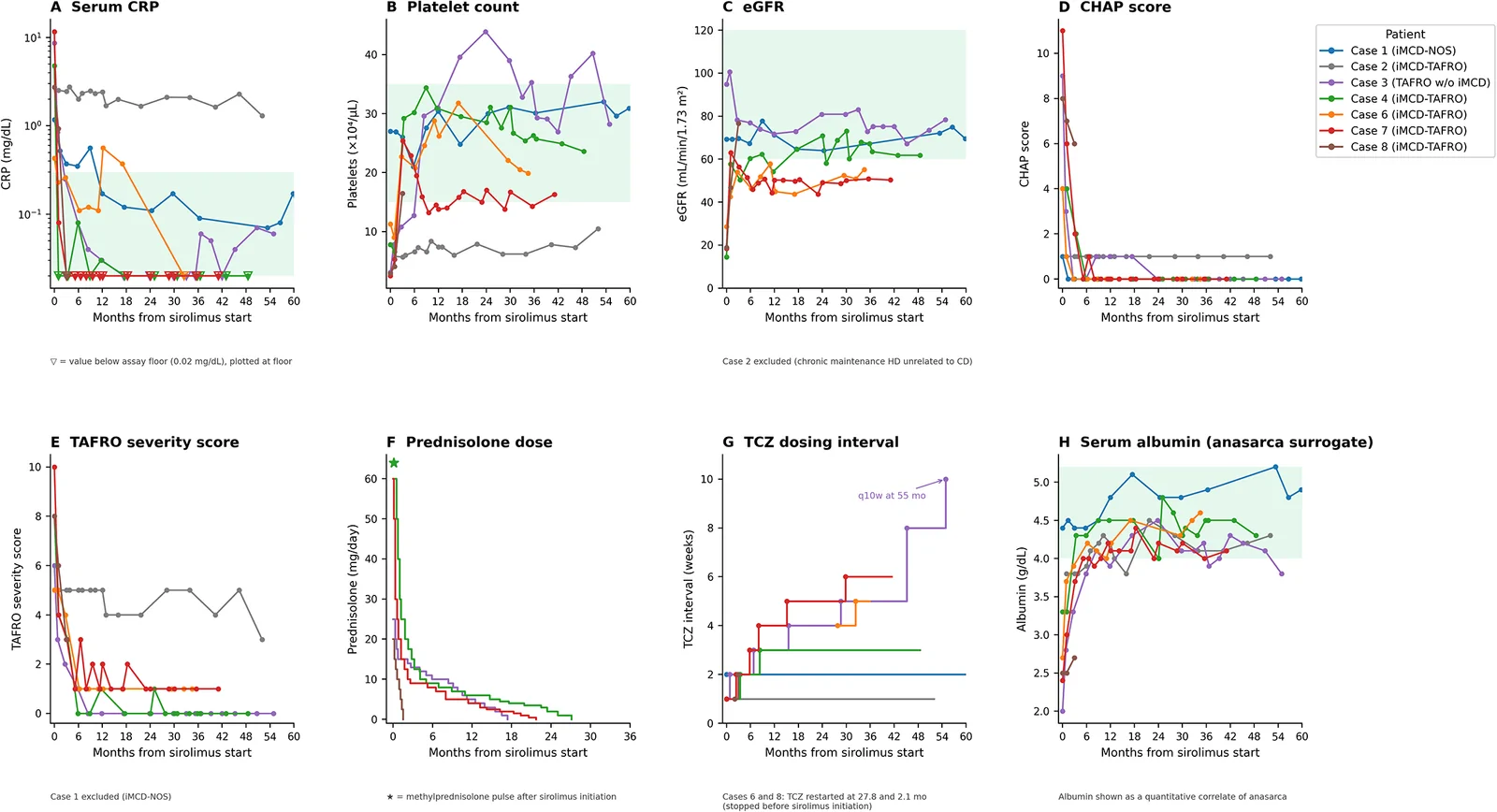
Clinical and laboratory course on add-on sirolimus in the seven patients with longitudinal data (Case 5 was followed at another institution after 0.7 months). The x-axis shows months from sirolimus initiation (0–60 months, ticks every 6 months; panel F, 0–36 months). Shaded zones indicate normal ranges. **(A–C)** Serum CRP (log scale; open triangles mark values below the assay floor of 0.02 mg/dL), platelet count (×10^4^/µL) and eGFR (mL/min/1.73 m²); Case 2 is excluded from the eGFR panel because of chronic maintenance hemodialysis unrelated to Castleman disease. **(D)** CHAP score. **(E)** TAFRO severity score (Case 1, iMCD-NOS, excluded). **(F)** Prednisolone maintenance dose; stars mark methylprednisolone pulses given after sirolimus initiation. **(G)** Tocilizumab dosing interval; in Cases 6 and 8 tocilizumab had been stopped before sirolimus initiation and was reintroduced at 27.8 and 2.1 months, respectively. **(H)** Serum albumin as a quantitative correlate of anasarca.

CHAP and TAFRO severity scores are reported for the same analysis set, the six patients with active TAFRO-phenotype disease at sirolimus initiation (Cases 2, 3, 4, 6, 7 and 8). In this group the median baseline CHAP score was 6 (range, 1–11) and the median baseline TAFRO severity score was 7 (range, 5–10). Among the four responders in this group who were evaluable at 12 months (Cases 3, 4, 6 and 7), the median CHAP score had fallen to 0 (range, 0–1) and the median TAFRO severity score to 1 (range, 0–2), and both scores remained low during subsequent follow-up (**Figures 2D, E**). Case 8, a responder who was not evaluable at 12 months, had CHAP and TAFRO severity scores of 6 and 3 at 3 months, from 8 and 8 at baseline. Case 2, the non-responder, had unchanged scores throughout (CHAP score 1 and TAFRO severity score 5 at every timepoint). Of the patients outside this group, Case 1 (iMCD-NOS) had a CHAP score of 1 at baseline and 0 from 1 month onward, and the TAFRO severity score is not applicable to this patient; Case 5, who was clinically inactive at initiation, had baseline CHAP and TAFRO severity scores of 0. Per-patient scores at baseline and at 3 and 12 months are given in **Table 2**.

Five of the eight patients (Cases 3, 4, 5, 7, and 8) were receiving prednisolone at sirolimus initiation (in Case 4, prednisolone 60 mg/day was started 6 days after sirolimus initiation, immediately after a methylprednisolone pulse, and this patient is counted among those receiving prednisolone). Among the four responders on prednisolone (Cases 3, 4, 7, and 8), glucocorticoids were tapered and discontinued in all four (**Figure 2F**); Case 5 discontinued sirolimus at 0.7 months (20 days) for adverse events and was subsequently followed at another institution on an unchanged prednisolone dose of 10 mg/day, so that prednisolone was withdrawn in four of the five patients who were receiving it. TCZ dosing intervals were extended to at least ≥5 weeks in three of the six responders, with a maximum interval of every 10 weeks in Case 3 (**Figure 2G**); per-timepoint prednisolone doses and TCZ intervals are given in **Table 2**. All six responders eventually discontinued sirolimus and had no relapse during available follow-up after discontinuation. Sirolimus exposure was 53.4, 33.0, 25.0, 29.3, 5.5 and 1.6 months in Cases 1, 3, 4, 6, 7, and 8, respectively; measured from the date of sirolimus discontinuation, post-discontinuation follow-up in the same patients was 30.1, 21.8, 23.5, 6.7, 35.9, and 1.4 months (median, 22.7 months; range, 1.4–35.9 months). Post-discontinuation observation in Case 8 was limited to 1.4 months because of transfer to another institution.

With the prespecified stringent response definition (CRP normalization plus platelet recovery and/or improvement in renal function or anasarca, with no escalation of concomitant immunosuppression), response proportions were lower than under the primary definition: 3 of 5 (60%; 95% CI 15–95) at 3 months and 2 of 4 evaluable patients (50%; 95% CI 7–93) at 12 months in the efficacy set, and 4 of 6 (67%; 95% CI 22–96) and 3 of 5 evaluable (60%; 95% CI 15–95) in the broader TAFRO-phenotype group. Two patients were reclassified, each on a single criterion. Case 8 met every clinical and laboratory component at 3 months but received cyclosporine A between 1.8 and 2.0 months, after elective sirolimus discontinuation at 1.6 months prompted by concern for possible invasive pulmonary aspergillosis; although this represented substitution rather than intensification of immunosuppression, it violates the literal no-escalation component. Case 6 met all components at 3 months but recorded a transient CRP of 0.56 mg/dL at the 12-month visit (0.11 mg/dL at 11 months and 0.27 mg/dL at 13 months) and therefore did not meet the CRP-normalization component at that timepoint. The stringent definition therefore does reclassify individual patients, and the gap between the two sets of estimates illustrates how sensitive small-denominator proportions are to single measurements and to the handling of therapy changes. The complete criterion-by-criterion assessment for every patient at both timepoints, including which components were not applicable or not evaluable, is given in **Table 1**. Representative radiographic findings are shown in **Figure 3**.

**Figure 3:**
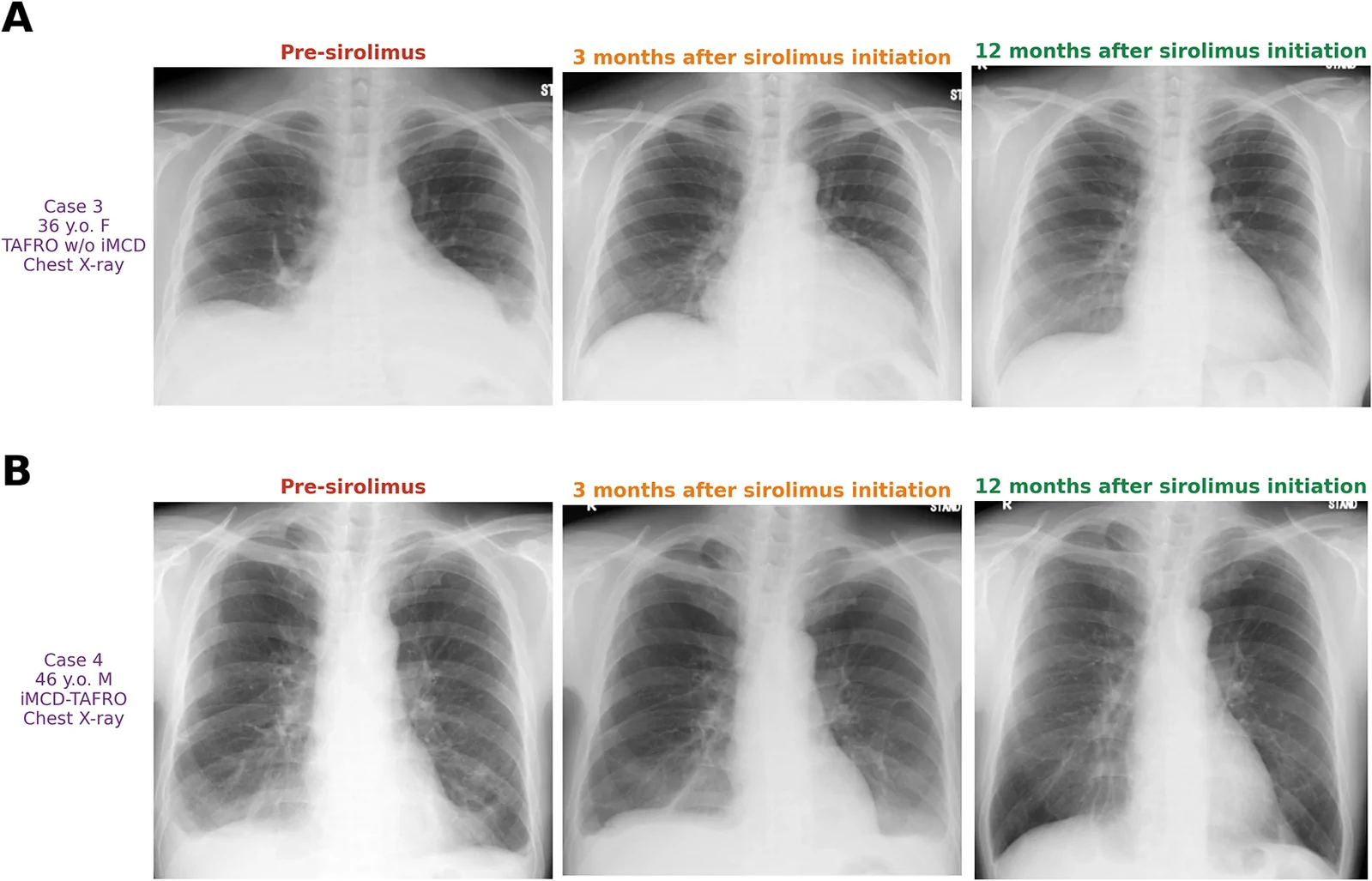
Radiographic response to add-on sirolimus in representative cases. Sequential chest X-rays are shown before sirolimus (active disease) and 3 and 12 months after sirolimus initiation. **(A)** Case 3 (36-year-old female, TAFRO syndrome without iMCD): bilateral pleural effusion at active disease, improvement at 3 months and resolution at 12 months. **(B)** Case 4 (46-year-old male, iMCD-TAFRO): bilateral pleural effusion at active disease, improvement at 3 months and resolution at 12 months.

### 3.2 Safety

During the observation period of this series, adverse events occurred in two of the eight patients (Cases 2 and 5). Every event, its time to onset from sirolimus initiation, the sirolimus dose at onset, the action taken, the outcome, and the investigator-assigned attribution are given in **Table 4**. In addition, Case 1 had discontinued an earlier course of sirolimus for adverse events before the observation period began, as described below.

**Table 4:** Adverse events during add-on sirolimus in the eight-patient safety cohort.

Case	Subtype	Analysis cohort	Adverse event	Time to onset from sirolimus initiation	Sirolimus dose at onset	Action taken	Outcome	Attribution to sirolimus
1	iMCD-NOS	Descriptive	None during the observation period (prior intolerance to 2 mg/day; see footnote)	—	—	—	—	—
2	iMCD-TAFRO	Efficacy	Fever; lymphocytopenia	43 days (1.4 months)	1 mg/day	Sirolimus permanently withdrawn	Resolved	Related
3	TAFRO w/o iMCD	Secondary	None reported	—	—	—	—	—
4	iMCD-TAFRO	Efficacy	None reported	—	—	—	—	—
5	iMCD-TAFRO	Safety only	Recurrent oral stomatitis; peripheral edema	7 days (0.2 months)	1 mg/day	Sirolimus permanently withdrawn	Resolved	Related
6	iMCD-TAFRO	Efficacy	None reported	—	—	—	—	—
7	iMCD-TAFRO	Efficacy	None reported	—	—	—	—	—
8	iMCD-TAFRO	Efficacy	None reported	—	—	—	—	—

Adverse events occurred in 2 of 8 patients (Cases 2 and 5) during the observation period. Both events were non-serious, occurred within the first 6 weeks of exposure, resolved completely, and were judged by the treating physicians to be related to sirolimus; sirolimus was permanently withdrawn in both patients. No infection requiring hospitalization, no opportunistic infection, no malignancy and no death occurred during sirolimus exposure in any patient.

Case 1 had been started on sirolimus 2 mg/day before the observation period of this series and developed rash and recurrent oral stomatitis 10 days after that start; sirolimus was withdrawn and the events resolved. Sirolimus was subsequently restarted at 1 mg every other day, which is the initiation date used throughout this series, and no adverse event occurred during the ensuing 53.4 months of treatment.

Case 8 discontinued sirolimus at 1.6 months because of clinical concern for possible invasive pulmonary aspergillosis. This was a pre-emptive discontinuation on suspicion; invasive aspergillosis was not confirmed microbiologically or histologically, no antifungal-treated infection was recorded as an adverse event, and the event is therefore not listed above as a sirolimus-attributed adverse event. Rituximab had also been interrupted for the same concern 0.3 months before sirolimus initiation.

Time to onset is the interval from the first dose of sirolimus to the first documentation of the event.

Attribution was assigned by the treating physicians and was not adjudicated independently.

Shaded rows indicate the two patients in whom an adverse event occurred during the observation period (Cases 2 and 5).

Both events occurred early, within the first 6 weeks of exposure. Case 2 developed fever and lymphocytopenia 43 days (1.4 months) after initiation while receiving 1 mg/day; sirolimus was permanently withdrawn and the events resolved. Case 5 developed recurrent oral stomatitis and peripheral edema 7 days after initiation; sirolimus was permanently withdrawn and both events resolved. Both events were judged by the treating physicians to be related to sirolimus, and both resolved completely. Case 1 had previously been started on sirolimus at 2 mg/day, before the observation period of this series, and developed rash and recurrent oral stomatitis 10 days after that start; sirolimus was withdrawn and the events resolved. Sirolimus was subsequently restarted at 1 mg every other day, which is the initiation date used throughout this series, and no adverse event occurred during the ensuing 53.4 months of treatment. No grade 4 adverse event, no infection requiring hospitalization, no opportunistic infection, no malignancy and no death occurred during sirolimus exposure in any patient. Separately, in Case 8 sirolimus was electively discontinued at 1.6 months because of clinical concern about possible invasive pulmonary aspergillosis arising during a treatment course that also included rituximab and high-dose glucocorticoids; rituximab had been interrupted for the same concern 0.3 months before sirolimus was started. Invasive aspergillosis was not confirmed microbiologically or histologically and no antifungal-treated infection was recorded, so this pre-emptive discontinuation is not listed in **Table 4** as a sirolimus-attributed adverse event. Clinical response was preserved at the final on-site assessment before transfer to another institution.

### 3.3 Exploratory paired single-cell RNA sequencing in one TAFRO-phenotype patient (hypothesis-generating)

To explore immune changes associated with sirolimus in the TAFRO-subtype, we performed paired single-cell RNA sequencing of peripheral blood mononuclear cells (PBMCs) from one TAFRO-phenotype patient (Case 3, TAFRO syndrome without iMCD) at diagnosis, before any treatment, and 3 months after sirolimus initiation, while the patient was receiving sirolimus together with tocilizumab and prednisolone (Methods §2.3). The differences between the two samples therefore reflect the combined treatment regimen and clinical recovery, not sirolimus alone. This analysis was part of the secondary TAFRO-phenotype cohort and was not intended to provide formal patient-level statistical inference; results should be regarded as exploratory within-patient biological changes in a single representative case.

After quality control, 14,839 cells (active disease, 6,251; sirolimus treatment, 8,588) were retained and grouped into 17 Leiden clusters, which were assigned to canonical PBMC lineages: CD4 T cells, CD8 T cells, NK cells, B cells, monocytes, plasma cells, plasmacytoid dendritic cells, platelets and erythrocytes (**Figure 4A**). The top 15 marker genes for every cluster, the lineage assigned to it and the basis for that assignment are given in **Supplementary Table S1**. Three clusters carried no lineage-defining marker among their top-ranked genes and were assigned from CD3D/CD3E positivity together with the presence or absence of CD8A and CD8B, and one further cluster (KLRB1, GZMK, SPOCK2, RORA, IL7R) had an intermediate phenotype; the CD4/CD8 boundary therefore depends in part on how these clusters are handled. To test the robustness of the composition, we independently re-derived it from the deposited count matrices during revision: monocyte, B-cell, NK-cell, platelet and erythrocyte counts were reproduced to within 20 cells at each timepoint, and the direction and approximate magnitude of the CD4 and CD8 changes were also reproduced (**Supplementary Table S1**). Cellular composition changed after sirolimus treatment (**Figures 4B, C**): inflammatory CD14+ monocytes decreased from 7.7% to 2.3%, and B cells decreased from 4.8% to 2.0%. In contrast, NK cells increased from 15.4% to 21.7%, and CD8 T cells increased from 26.3% to 34.1%, whereas CD4 T-cell proportions were largely unchanged. Because peripheral proportions are compositional and may also be influenced by sample handling, these shifts are reported descriptively.

**Figure 4:**
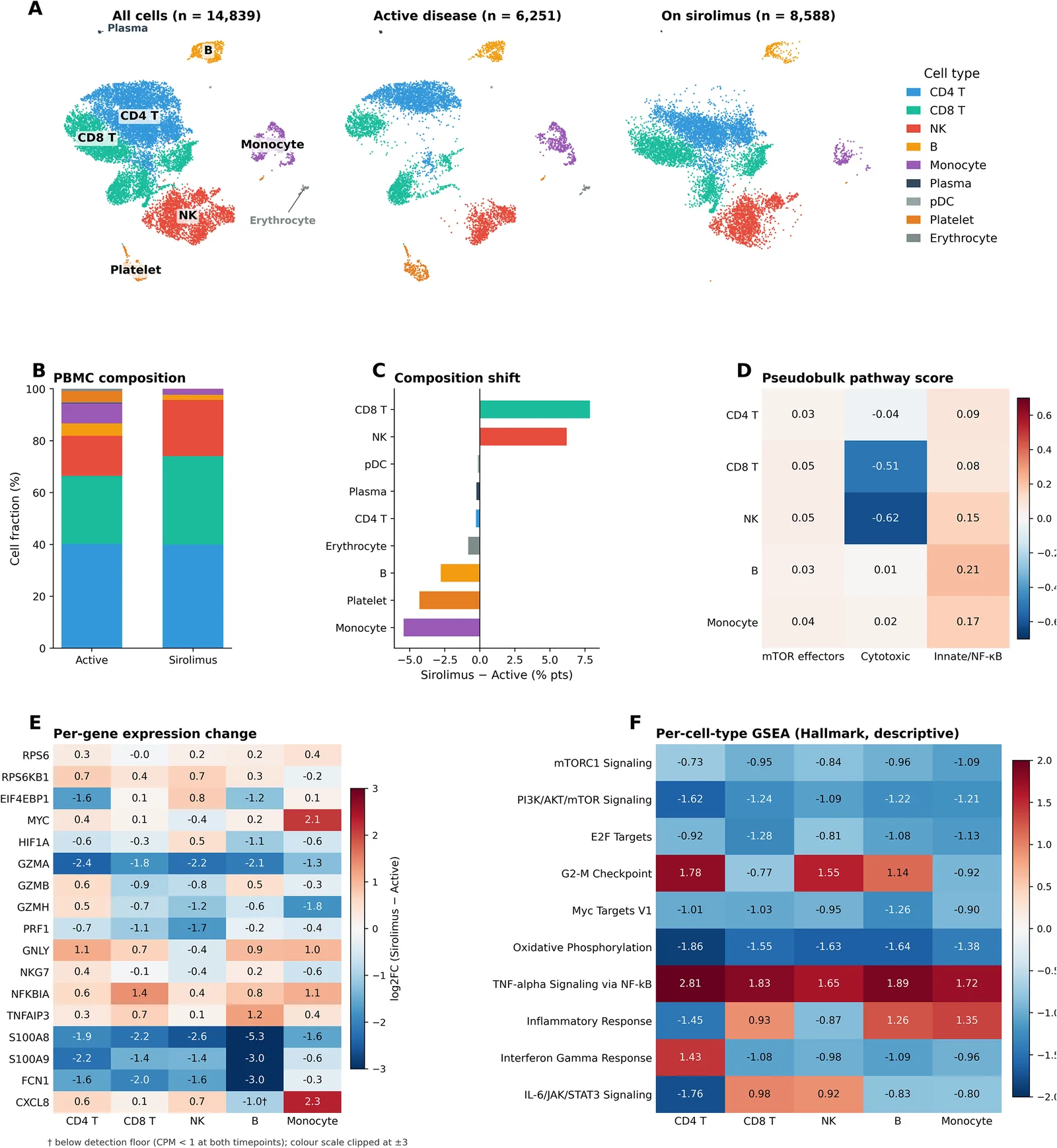
Exploratory paired single-cell RNA-sequencing of PBMCs from one TAFRO-phenotype patient (Case 3, TAFRO syndrome without iMCD) at diagnosis before any treatment (“active”) and 3 months after sirolimus initiation, while receiving sirolimus together with tocilizumab and prednisolone (“sirolimus”); findings are hypothesis-generating for the TAFRO-subtype (n=1). **(A)** UMAP embedding of 14,839 high-quality cells colored by cell type, shown combined and split by time-point; cell-type labels are placed on the combined UMAP, and the 17 Leiden clusters underlying the nine cell types are listed in **Supplementary Table S1**. **(B)** Stacked bar of PBMC composition. **(C)** Per-cell-type compositional shift on sirolimus (Sirolimus − Active, percentage points). **(D)** Pseudobulk per-cell-type pathway-score change (Sirolimus − Active) for three biology-anchored signatures; negative values indicate decrease on sirolimus. The mTOR-component signature shows essentially no change at the transcript level, consistent with the post-translational mode of mTORC1 regulation. **(E)** Heatmap of per-gene log2 fold change (Sirolimus − Active) by cell type for the same signatures, showing clear suppression of cytotoxic-effector and proliferation genes in NK and CD8 T cells; the color scale is clipped at ±3, annotated values are unclipped, and a dagger marks genes below the detection floor (CPM <1 at both timepoints). **(F)** Per-cell-type Hallmark GSEA preranked analysis (gene sets ranked by Sirolimus − Active log2 fold change) for five cell populations (CD4 T, CD8 T, NK, B, monocyte), showing normalized enrichment scores (NES). Negative NES = pathway suppressed on sirolimus; positive NES = pathway relatively enriched on sirolimus. NES, FDR q-values, and full leading-edge gene lists are provided in **Supplementary Table S3**.

Per-cell-type pseudobulk and gene-level fold-change analyses showed lower expression of cytotoxic-effector genes, including GZMA, GZMB, GZMH, PRF1, GNLY, and NKG7, in NK-cell and CD8 T-cell populations. Proliferation-associated genes, including MKI67, CDCA7, PCLAF, and TYMS, were also reduced. By contrast, the transcriptional mTOR-component/effector signature (mTOR, RPS6, EIF4EBP1, EIF4E, MYC, and HIF1A) changed little at the transcript level, consistent with the predominantly post-translational regulation of mTORC1 activity (**Figures 4D, E**). Pseudobulk fold changes were computed on counts-per-million–normalized summed counts with an explicit pseudocount of 1 CPM; genes below a detection floor of 1 CPM in both timepoints are flagged, and the underlying cell numbers, library sizes and CPM values for every gene and cell type are provided in **Supplementary Table S2**. The B-cell compartment is represented by relatively few cells (299 at active disease and 171 on sirolimus), and gene-level estimates in this compartment should therefore be interpreted with corresponding caution.

Hallmark gene-set enrichment analysis of the preranked per-cell-type gene lists showed that oxidative phosphorylation was the only program consistently and nominally significantly suppressed, reaching a nominal false-discovery rate below 0.05 in CD4 T-cell, CD8 T-cell, NK-cell and B-cell populations (**Figure 4F**). Reductions in mTORC1 signaling, PI3K/AKT/mTOR signaling, Myc targets V1 and E2F targets were directionally concordant across cell types but did not reach nominal significance. The TNF-α via NF-κB and inflammatory response signatures were not suppressed and instead showed positive enrichment scores; inspection of the leading-edge genes showed that this signal was driven by NF-κB negative-feedback regulators (NFKBIA, TNFAIP3, DUSP1/2, NFKB2, BCL3) and immediate-early stress-response genes (GADD45B, IER3, KLF6, PPP1R15A), rather than by canonical pro-inflammatory effectors, consistent with the per-gene increases in NFKBIA and TNFAIP3 shown in **Figure 4E**. Because this analysis compares a single sample at each timepoint, the permutation statistics test gene-set coherence within a ranked list rather than reproducibility across biological replicates; the enrichment scores are therefore presented as descriptive, and no significance annotation is shown on **Figure 4F**. Nominal p and q values are reported in **Supplementary Table S3** for completeness only.

Additional module-score analyses and lineage-restricted analyses are presented in **Supplementary Figure S1**. Monocyte sub-clustering analyses are shown in **Supplementary Figure S2**.

## 4 Discussion

In this case series, add-on sirolimus showed clinically meaningful activity in refractory iMCD-TAFRO: 4 of 5 patients in the efficacy set responded at 3 months (80%; 95% CI 28–99) and 3 of 4 evaluable patients sustained response at 12 months (75%; 95% CI 19–99). A broader TAFRO-phenotype analysis that included Case 3 (TAFRO syndrome without iMCD) showed similar proportions (5 of 6, 83%; 95% CI 36–100, at 3 months; 4 of 5 evaluable, 80%; 95% CI 28–99, at 12 months). Under the prespecified stringent definition these proportions fell to 3 of 5 (60%) and 2 of 4 evaluable (50%) in the efficacy set, because two patients failed a single component each — a post-discontinuation switch to cyclosporine A in Case 8 and an isolated transient CRP rise in Case 6. Because these denominators are five and six patients, the confidence intervals span most of the plausible range, a single reclassification moves the point estimate by 20 percentage points or more, and the estimates are hypothesis-generating rather than precise. Responses occurred across a broad severity range, including severe TAFRO manifestations and dialysis-dependent renal failure. Treatment was also associated with clinically important treatment-sparing outcomes, including glucocorticoid discontinuation, extension of TCZ dosing intervals, and withdrawal from hemodialysis. Because anasarca can be slow to resolve, persistent or slowly resolving anasarca in otherwise improving patients (e.g., Case 6, whose anasarca sub-score remained at its baseline value through month 3 and reached zero by month 6) should not, on its own, be interpreted as treatment failure. We also note that combining iMCD-TAFRO with TAFRO syndrome without iMCD in the secondary analysis merges two nosological entities; this grouping is presented only as a prespecified secondary analysis on the basis of their shared TAFRO clinical phenotype and overlapping treatment approach, and the primary efficacy set is restricted to iMCD-TAFRO.

A key distinguishing feature of this study is the composition of the efficacy analysis. The largest published sirolimus study consisted mainly of iMCD-NOS cases, with only 2 of 26 patients (7.7%) classified as iMCD-TAFRO (10). By contrast, all five patients in our efficacy analysis had active refractory iMCD-TAFRO. Given the severe clinical profile of the TAFRO-subtype, including thrombocytopenia, anasarca, renal dysfunction, and inadequate anti–IL-6 responses, the 80% response rate at 3 months suggests that sirolimus may be particularly useful in this phenotype, though as an adjunct to standard treatment.

These findings are consistent with prior evidence supporting mTOR inhibition in refractory iMCD. Fajgenbaum and colleagues reported clinical benefit in three patients with anti–IL-6–refractory iMCD and PI3K/Akt/mTOR pathway activation, (5) and a retrospective Chinese study found symptomatic and biochemical responses in 18 of 26 patients receiving sirolimus-containing regimens (10). Our data extend these observations to a TAFRO-enriched cohort with severe clinical manifestations.

Durable disease control after sirolimus discontinuation was another important observation. In previous studies, many patients remained on sirolimus at the time of analysis or continued treatment during follow-up. (10, 11) In our cohort, all six responders eventually discontinued sirolimus yet had no relapse during available follow-up after discontinuation (median, 22.7 months; range, 1.4–35.9 months, measured from the date of sirolimus discontinuation; **Table 2**). Four of the six responders had more than 20 months of documented off-drug follow-up. These results raise the possibility that, in selected patients with refractory iMCD-TAFRO, sirolimus may serve as finite-duration adjunctive induction or bridge therapy rather than indefinite maintenance therapy, although this hypothesis requires prospective testing. The feasibility of treatment discontinuation in selected responders is supported by a recent multicenter retrospective study of 27 patients with iMCD-TAFRO in biochemical complete response, 85.2% of whom maintained biochemical complete response after treatment withdrawal at a median follow-up of 31 months; however, that study did not specifically evaluate sirolimus, and most patients had received myeloma-like treatment. (23) We note that this observation concerns disease status after withdrawal of a drug that had already produced a response, and is therefore distinct from the response assessments themselves; the treatment state at each assessment is given in **Table 2**.

Glucocorticoid dependence is a major challenge in iMCD-TAFRO. Prolonged exposure increases the risk of infection, osteoporosis, metabolic dysfunction, and cardiovascular disease. (24) Five of the eight patients were receiving prednisolone at sirolimus initiation, and prednisolone was withdrawn in the four responders among them; the fifth (Case 5) stopped sirolimus after 20 days and was immediately transferred to follow-up at another institution on an unchanged prednisolone dose of 10 mg/day. TCZ dosing intervals were also extended in several patients without apparent loss of disease control, and both patients with dialysis-dependent active disease discontinued hemodialysis after sirolimus initiation. These outcomes suggest potential glucocorticoid-, biologic-, and supportive-care-sparing effects of add-on sirolimus. We emphasize, however, that these outcomes cannot be attributed to sirolimus alone. All patients were receiving concomitant therapy at sirolimus initiation — tocilizumab in six, rituximab in two, prednisolone in five, and cyclosporine A in one — and several interventions overlapped closely in time with the observed improvements. In particular, hemodialysis was withdrawn 9 days (Case 7) and 21 days (Case 8) after sirolimus initiation, intervals during which concurrently administered agents may plausibly have contributed; Case 8 in particular received rituximab infusions at 0.5 and 0.7 months and high-dose glucocorticoids over the same period, and Case 6 received rituximab infusions at 0.1 and 0.4 months as the completion of a course begun before sirolimus was started. Per-patient concomitant therapy timelines are shown in **Figure 1** and the treatment state at each assessment in **Table 2**, so that readers can assess attribution directly. One patient (Case 6) had an extended period of sirolimus without concurrent anti–IL-6 therapy: rituximab was given immediately before and just after sirolimus initiation, sirolimus was then continued without tocilizumab for 27.8 months, and tocilizumab was reintroduced every 4 weeks at that point and extended to every 5 weeks before sirolimus was withdrawn at 29.3 months. This single sequence is the one instance in the series in which sirolimus was given without concurrent anti–IL-6 therapy for a prolonged period; it does not constitute evidence for sirolimus monotherapy as a treatment strategy, particularly as it followed a full rituximab course, and the remaining patients were all treated with sirolimus added to ongoing standard therapy.

The paired single-cell analysis in one TAFRO-phenotype patient (TAFRO syndrome without iMCD) suggested coordinated, treatment-associated reductions in cytotoxic-effector and proliferation-associated transcriptional programs and in oxidative phosphorylation, with directionally concordant but non-significant reductions in mTORC1-, PI3K/AKT/mTOR-, Myc- and E2F-related Hallmark gene sets. The Hallmark TNF-α via NF-κB and inflammatory response signatures were not suppressed and showed relative enrichment on sirolimus; the leading-edge genes driving this signal were dominated by NF-κB negative-feedback regulators (NFKBIA, TNFAIP3, NFKB2, BCL3, DUSP1/2) and immediate-early stress-response genes (GADD45B, IER3, KLF6, PPP1R15A, FOS, JUN) rather than canonical pro-inflammatory effectors. Per-gene fold-change analysis also showed increases in NFKBIA and TNFAIP3 across PBMC populations after sirolimus exposure (**Figure 4E**). These observations are more consistent with engagement of an NF-κB negative-feedback and cellular stress-response program than with paradoxical pro-inflammatory NF-κB activation. TNF signaling has separately been proposed as a candidate pathway in anti–IL-6–refractory iMCD on the basis of a multi-omic analysis and a single proof-of-concept case treated with adalimumab, (7) so the behavior of TNF-responsive genes during sirolimus treatment merits attention in larger datasets. They are also convergent with our own longitudinal serum proteomic sub-analysis of a sirolimus-treated TCZ-resistant iMCD patient, which reported suppression of IL-6/JAK-STAT3, TNF-α/NF-κB, IFN-γ and chemokine signaling. (18) Accordingly, we do not claim that longitudinal immune profiling during sirolimus treatment is itself novel; rather, the present dataset adds cell-type-resolved, single-cell resolution to that earlier bulk-proteomic observation, and the concordance between the two orthogonal platforms strengthens both. Because the present analysis was limited to a single patient without iMCD, and because the on-treatment sample was obtained while the patient was also receiving tocilizumab and prednisolone — so that the transcriptional differences reflect the combined regimen and clinical recovery rather than sirolimus alone — the findings remain preliminary and hypothesis-generating for the broader TAFRO-phenotype spectrum, including iMCD-TAFRO, and larger transcriptional studies are needed.

Our observations also align qualitatively with recent peripheral profiling studies in iMCD. Plasma proteomic analyses identified a “chemokine storm” characterized by activation of CXCL chemokine and complement-related pathways, (25) consistent with broader peripheral inflammatory dysregulation beyond IL-6 alone. Chan and colleagues described inflammatory monocytosis characterized by S100-family gene expression, (26) and a single-cell study of peripheral blood from 15 patients with iMCD identified an IFN-γ–driven axis in which CCL-expressing inflammatory monocytes, the cells with the strongest IL-6 signaling activation, interact with NK/NKT cells during flares, with serum IFN-γ correlating with disease severity and treatment resistance. (27) At the tissue level, integrated bulk and spatial proteomics of iMCD-TAFRO lymph nodes has identified angiogenesis, interferon signaling, complement, fibrosis, and macrophage-associated signatures across distinct tissue compartments, (28) suggesting that the peripheral changes we observed may reflect a broader, tissue-based vascular–fibroinflammatory process. In our paired comparison, inflammatory monocytes and B-cell–associated transcriptional programs decreased after sirolimus treatment, and pathway analyses showed reductions in mTORC1- and proliferation-related signatures. Mechanistic interpretation should remain cautious because this analysis was exploratory and limited to a single patient.

This study has several limitations. First, it was a small, single-center observational study with heterogeneous concomitant treatments, and with five and six patients in the efficacy analyses the confidence intervals around the response proportions are very wide. Second, the response proportions are sensitive to the definition applied: the primary and stringent definitions differ by 20 percentage points at 3 months and 25 percentage points at 12 months in the efficacy set, driven by a single component in a single patient at each timepoint, so no single headline proportion should be read as a stable estimate of efficacy (**Table 1**). Third, in the absence of a control group and given that every patient received concomitant therapy, the contribution of sirolimus cannot be isolated; the concomitant-therapy timelines in **Figure 1** are provided so that readers can judge attribution, and several improvements — notably the withdrawal of hemodialysis on days 9 and 21 in Cases 7 and 8 — occurred over intervals during which other agents were also active. Fourth, the transcriptomic analysis was based on paired samples from a single representative TAFRO-phenotype patient (TAFRO syndrome without iMCD), whose pre-treatment sample was obtained before any therapy and whose on-treatment sample was obtained during combined sirolimus, tocilizumab and prednisolone, so that the differences cannot be attributed to sirolimus alone; it is exploratory, its permutation statistics do not test reproducibility across biological replicates, and it requires confirmation in larger iMCD-TAFRO cohorts. Fifth, the CD4/CD8 T-cell boundary depends in part on the handling of clusters that lack lineage-defining markers; an independent re-derivation from the deposited matrices reproduced the reported composition, but the split should still be read as approximate (**Supplementary Table S1**). The B-cell compartment also contained relatively few cells, so per-gene estimates in that compartment are imprecise. Sixth, sirolimus therapeutic drug monitoring was not routinely available, precluding exposure–response analyses. Finally, response assessment was retrospective and unblinded, and clinical data for Case 5 were available only at baseline.

In conclusion, in this small TAFRO-enriched series, add-on sirolimus was associated with clinical improvement in refractory iMCD-TAFRO, including in patients with severe TAFRO manifestations, and with treatment-sparing outcomes such as glucocorticoid withdrawal, extension of biologic dosing intervals, and discontinuation of hemodialysis, which were sustained after sirolimus was stopped. Because all patients received concomitant standard therapy and the denominators are small, these observations support sirolimus only as an adjunctive rescue option added to standard treatment (anti–IL-6 therapy and, where appropriate, glucocorticoids), and not as a substitute for established first-line therapy; sirolimus monotherapy is not supported by these data. Its role as a finite-duration adjunctive rescue strategy warrants prospective evaluation in larger studies.

## Data Availability

The single-cell RNA-sequencing datasets generated and analyzed in this study have been deposited in the NCBI Gene Expression Omnibus (GEO) under accession number GSE329476 (https://www.ncbi.nlm.nih.gov/geo/query/acc.cgi?acc=GSE329476). Analysis code is available from the corresponding author on reasonable request.
