# Supplementary material for: Add-on sirolimus in refractory iMCD-TAFRO: a TAFRO-enriched 8-patient case series with paired single-cell profiling
Source: Front Immunol. 2026 Oct 16;17:1963440. doi: 10.3389/fimmu.2026.1963440 (PMC13719344; doi:10.3389/fimmu.2026.1963440)
Supplement: Supplementary file 1 [file DataSheet1.pdf]

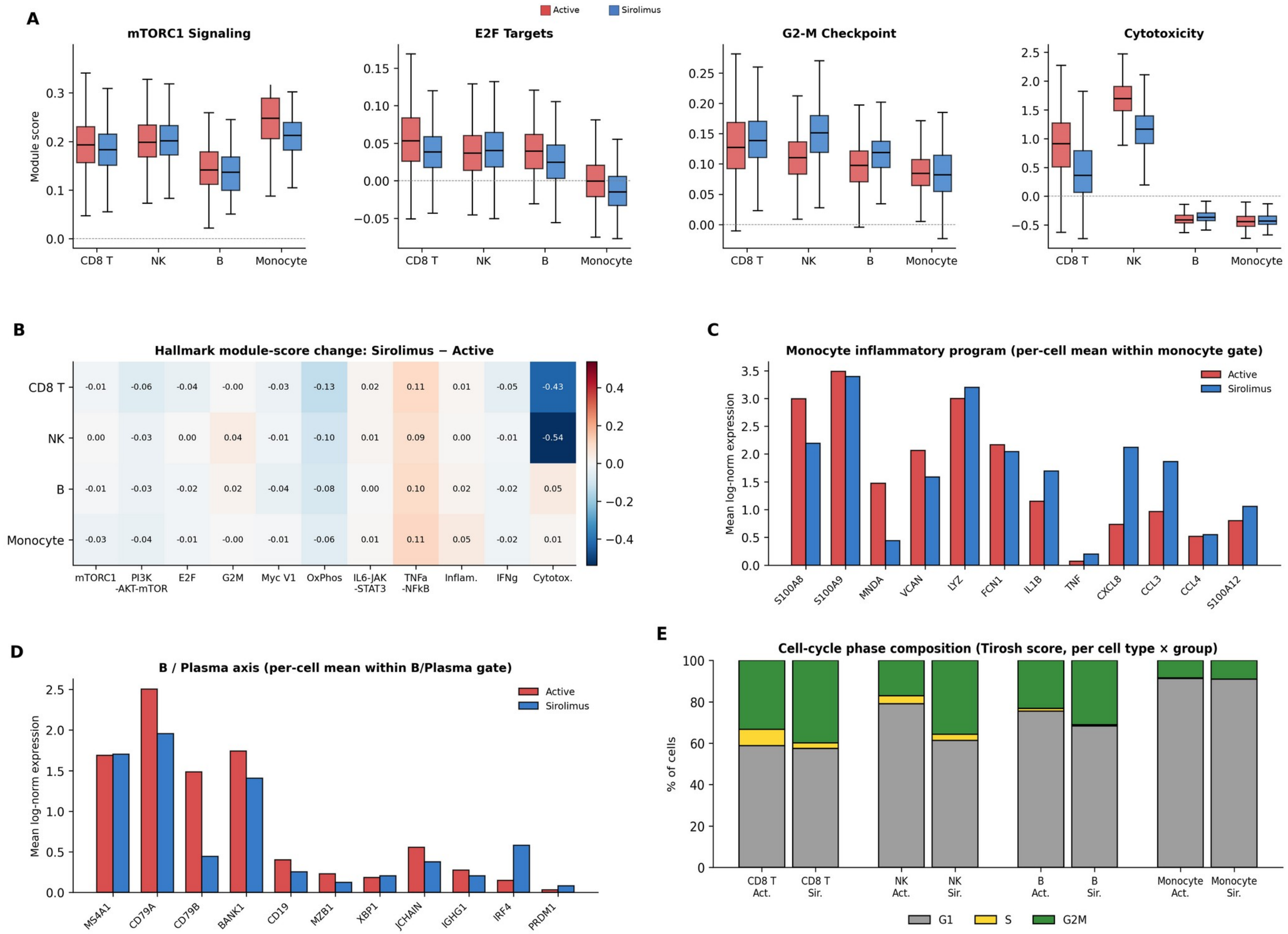

Supplementary Figure S1. Module-score and sub-population analyses of paired peripheral scRNA-seq from Case 3. (A) Boxplots of per-cell Hallmark mTORC1, E2F-targets, G2-M-checkpoint and curated cytotoxicity module scores in CD8 T, NK, B and monocyte compartments at active disease (red) versus on sirolimus (blue). (B) Heatmap of mean per-cell module-score change (Sirolimus − Active) for 11 Hallmark/curated signatures across the four lineages. (C) Mean log-normalized expression of canonical inflammatory-monocyte markers within the monocyte gate. (D) Mean log-normalized expression of B/plasma-axis markers within the B/plasma gate. (E) Cell-cycle phase composition (G1, S, G2-M; Tirosh score) per cell type and group, demonstrating that within-lineage cell-cycle distribution is comparable between active disease and on sirolimus.

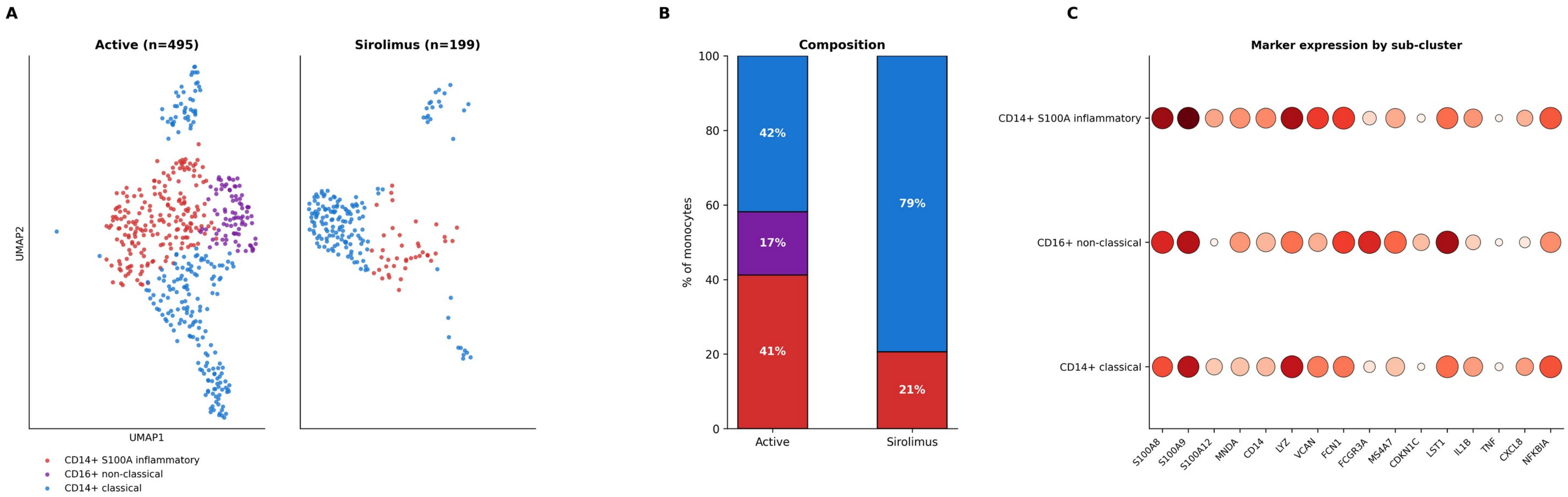

Supplementary Figure S2. Inflammatory monocyte sub-clustering of paired peripheral scRNA-seq from Case 3. (A) UMAP of the 694 monocytes faceted by group, colored by sub-cluster (CD14+ S100A inflammatory, CD14+ classical, CD16+ non-classical). (B) Stacked bar of sub-cluster composition at active disease versus on sirolimus, demonstrating selective contraction of the CD14+ S100A inflammatory and CD16+ non-classical subsets and relative expansion of the CD14+ classical subset on sirolimus. (C) Dotplot of canonical monocyte sub-cluster markers; dot color = mean log-normalized expression, dot size = percent of cells expressing the gene.
